# Delta-Catenin as a Modulator of Rho GTPases in Neurons

**DOI:** 10.3389/fncel.2022.939143

**Published:** 2022-07-04

**Authors:** Maxsam S. Donta, Yogesh Srivastava, Pierre D. McCrea

**Affiliations:** ^1^Department of Genetics, The University of Texas MD Anderson Cancer Center, Houston, TX, United States; ^2^Program in Genetics and Epigenetics, The University of Texas MD Anderson Cancer Center University of Texas Health Science Center Houston Graduate School of Biomedical Science, Houston, TX, United States; ^3^Program in Neuroscience, The University of Texas MD Anderson Cancer Center University of Texas Health Science Center Houston Graduate School of Biomedical Science, Houston, TX, United States

**Keywords:** delta-catenin, Rho GTPase, RhoA, Rac1, Cdc42, catenin, GTPase, neurons

## Abstract

Small Rho GTPases are molecular switches that are involved in multiple processes including regulation of the actin cytoskeleton. These GTPases are activated (turned on) and inactivated (turned off) through various upstream effector molecules to carry out many cellular functions. One such upstream modulator of small Rho GTPase activity is delta-catenin, which is a protein in the p120-catenin subfamily that is enriched in the central nervous system. Delta-catenin affects small GTPase activity to assist in the developmental formation of dendrites and dendritic spines and to maintain them once they mature. As the dendritic arbor and spine density are crucial for synapse formation and plasticity, delta-catenin’s ability to modulate small Rho GTPases is necessary for proper learning and memory. Accordingly, the misregulation of delta-catenin and small Rho GTPases has been implicated in several neurological and non-neurological pathologies. While links between delta-catenin and small Rho GTPases have yet to be studied in many contexts, known associations include some cancers, Alzheimer’s disease (AD), Cri-du-chat syndrome, and autism spectrum disorder (ASD). Drawing from established studies and recent discoveries, this review explores how delta-catenin modulates small Rho GTPase activity. Future studies will likely elucidate how PDZ proteins that bind delta-catenin further influence small Rho GTPases, how delta-catenin may affect small GTPase activity at adherens junctions when bound to N-cadherin, mechanisms behind delta-catenin’s ability to modulate Rac1 and Cdc42, and delta-catenin’s ability to modulate small Rho GTPases in the context of diseases, such as cancer and AD.

## Introduction

Small Rho GTPases are enzymes that bind and hydrolyze GTP. They act as molecular on-off switches by cycling between their GTP-bound active state and their GDP-bound inactive state through their interactions with a wide variety of regulatory and effector molecules. While GTPases are involved in almost all cellular processes, the Rho subfamily is best known for cytoskeletal reorganization. Small Rho GTPases have many modulators of their activity, with one being delta-catenin (Martinez et al., 2003; Abu-Elneel et al., 2008; Kim et al., 2008; Gu et al., 2009; Ghose et al., 2015). Delta-catenin is predominantly expressed in nervous tissue and is required for regular neuronal function (Lu et al., 1999; Ho et al., 2000). For example, delta-catenin affects the formation and maintenance of dendrites and dendritic spines, both of which are crucial for forming neuronal contacts and networks, and for synaptic plasticity and learning (Abu-Elneel et al., 2008; Yuan et al., 2015; McCann and Ross, 2017). As such, alterations of delta-catenin expression have been found in several neurological pathologies, including Alzheimer’s disease and Cri-du-chat syndrome. Many of delta-catenin’s functions are known or thought to be through its ability to modulate small Rho GTPase activity. Thus, delta-catenin’s ability to modulate small Rho GTPases is crucial for healthy neuronal function.

### Structure, Localization, and Expression of Delta-Catenin

**FIGURE 1:**
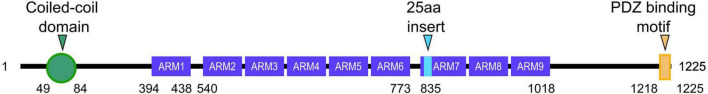
Schematic of delta-catenin protein structure. The structures include a coiled-coil domain (green), 9 armadillo (ARM) repeats (purple), and a PDZ binding motif (yellow). An alternative splicing insert that is 25 amino acids in length is in the 7th ARM repeat (cyan). The amino acid number corresponding to where each domain is listed below. Amino acids corresponding to small gaps between ARM domains are not shown.

Delta-catenin—also known as NPRAP or neurojungin (Paffenholz and Franke, 1997)—is a member of the p120-catenin protein subfamily, which also includes p120-catenin itself, ARVCF-catenin, and p0071-catenin. As is characteristic of the p120-subfamily, delta-catenin contains a central series of 10 Armadillo (ARM) repeats, with this Armadillo region being flanked by N- and C-terminal tail domains. Along with ARVCF and p0071, delta-catenin contains a PDZ-binding motif at its very C-terminus. In the brain, delta-catenin can additionally be found as a longer isoform that contains an extra coiled-coil within its N-terminal tail (Markham et al., 2014). While delta-catenin has four transcript variants, only the longest one contains the coiled-coil domain (Adegbola et al., 2020). A schematic of delta-catenin’s protein structure is shown in Figure 1. Unlike the other members of the p120-subfamily that are more uniformly expressed across mammalian tissues, delta-catenin is highly enriched in the central nervous system and retina (Lu et al., 1999; Ho et al., 2000). Delta-catenin has a splice variant that generates a 25-residue insert at the start of the 7th Armadillo domain (Paffenholz and Franke, 1997). While this variant is expressed throughout the fetal and adult brain, the shorter variant lacking this insert is predominant in most regions of the developing and mature brain (Kawamura et al., 1999). Currently, there are no known functional differences between these two isoforms (Ishiyama et al., 2010), but the location of the 25-residue insert on the longer isoform suggests a potential role in regulating the structure and function of the insert region (Yuan and Arikkath, 2017). Delta-catenin has established roles at adherens junctions where it stabilizes N-cadherin (Fannon and Colman, 1996; Benson and Tanaka, 1998; Israely et al., 2004; Yuan et al., 2015). It is also present in the cytoplasm and nucleus, even as delta-catenin’s roles in the latter are less certain. For example, while delta-catenin was found to bind the gene regulatory protein ZIFCAT in the nucleus upon delta-catenin’s caspase3-mediated cleavage, the functional extent of this interaction remains to be studied (Gu et al., 2011). Furthermore, uncleaved delta-catenin can enter the nucleus to bind and modulate the transcriptional regulator Kaiso (Rodova et al., 2004; Dai et al., 2011; McCrea and Gottardi, 2016). Delta-catenin also functionally interacts with the Wnt pathway, although it may not need to enter the nucleus itself in this context, where it promotes canonical Wnt/β-catenin/LEF-1-mediated transcription (Nopparat et al., 2015). Intriguingly, delta-catenin is also reported to be degraded by the Wnt-pathway destruction component Axin, suggesting some parallels with beta-catenin (Hong et al., 2010). In central neurons, immunocytochemistry has shown that delta-catenin is present in the cytosol and at the inner cell membrane and that it localizes to dendrites, postsynaptic terminals, and growth cones (Martinez et al., 2003; Arikkath et al., 2008; Yuan et al., 2015; Baumert et al., 2020).

### Small Rho GTPases

The Rho subfamily of small GTPases is part of the Ras superfamily of proteins. While more than 20 Rho GTPases are known to exist, the most fully characterized are RhoA (Ras homologous member A), Rac1 (Ras-related C3 botulinum toxin substrate 1), and Cdc42 (cell division cycle 42). GTPases are small G-proteins that bind to and hydrolyze guanosine triphosphate (GTP) to guanosine diphosphate (GDP). These GTPases cycle between their active GTP-bound states and their inactive GDP-bound states through the involvement of guanine nucleotide exchange factors (GEFs), GTPase-activating proteins (GAPs), and GDP dissociation inhibitors (GDIs). GEFs enhance the exchange of GDP for GTP to increase GTPase signaling, GAPs increase GTP hydrolysis to decrease GTPase signaling, and GDIs prevent the exchange of GDP for GTP to inhibit GTPase signaling (Zalcman et al., 1999; Hoffman et al., 2000; Schmidt and Hall, 2002; Bernards, 2003; Bernards and Settleman, 2004). Cycling between their active and inactive states allows for GTPases to regulate various biomolecular pathways that act upon the cytoskeleton. Among others, these pathways involve cell migration, cell adhesion, cell polarity, wound healing, membrane trafficking, and cytokinesis (Tapon and Hall, 1997; Heasman and Ridley, 2008; Hall, 2012; Zegers and Friedl, 2014; Arrazola Sastre et al., 2020; Clayton and Ridley, 2020). As such, Rho GTPases are involved in some capacity in all stages of neuron development, including regulating dendrite morphogenesis, dendritic spine dynamics, synaptic plasticity, neurotransmitter receptor clustering, long-term potentiation and neuronal survival (Jones et al., 2004; Auer et al., 2011; Martino et al., 2013; Musilli et al., 2013; Stankiewicz and Linseman, 2014; Vaghi et al., 2014; Hedrick and Yasuda, 2017; Zamboni et al., 2018). Functionally, these parameters in neuron development influence memory, learning, and cognition. Thus, misregulation of Rho GTPases leads to neurological impairments and pathologies (Guiler et al., 2021).

### RhoA

Small Rho GTPase activity can be experimentally modulated through the use of constitutively active (CA) and dominant negative (DN) mutations. As these GTPases have roles in actin reorganization, modulating their activity in various neuronal model systems has shown that small Rho GTPases play a substantial role in dendritic arbor development. RhoA tends to have an inhibitory effect on neuronal dendrites, as CA RhoA reduces dendritic arbor growth in mouse and rat hippocampal neurons, *Drosophila* mushroom body neurons, and *Xenopus* retinal ganglion cells (Ruchhoeft et al., 1999; Lee et al., 2000; Nakayama et al., 2000; Ahnert-Hilger et al., 2004; Pilpel and Segal, 2004). Conversely, DN RhoA increases the dendritic arbor in mouse and rat hippocampal neurons, overextends dendrites in *Drosophila* mushroom body neurons, and increases the dendritic arbor growth rate in *Xenopus* optic tectal neurons (Lee et al., 2000; Li et al., 2000; Ahnert-Hilger et al., 2004). Furthermore, RhoA activation causes growth cone collapse (Stankiewicz and Linseman, 2014). These studies collectively show that RhoA activation negatively regulates dendritic growth, and conversely that RhoA inhibition enhances it. Along with dendritic arborization, small Rho GTPases are known to play roles in dendritic spine morphology and maintenance. Similar to its negative regulation regarding dendrite arborization, CA RhoA reduces dendritic spine density in hippocampal neurons and slice cultures (Nakayama et al., 2000; Tashiro et al., 2000; Impey et al., 2010). While CA RhoA decreases spine length in mouse and rat hippocampal neurons, the effects of RhoA inhibition on spine morphology are less clear. DN RhoA increased spine length and reduced spine density in rat hippocampal neurons, increased spine density and spine length in mouse hippocampal neurons, and did not influence spine density in pyramidal neurons from rat hippocampal slices (Nakayama et al., 2000; Tashiro et al., 2000; Pilpel and Segal, 2004; Tashiro and Yuste, 2004; Impey et al., 2010; Murakoshi et al., 2011; Orefice et al., 2016; Zhang et al., 2021). These discrepancies may be caused by differences in neuron age, type, local levels of activity, definitions used for what constitutes dendritic spines, or distinctions in the extent of cell contact with other neurons or glia arising from non-equivalent plating densities (Govek et al., 2005). Overall, RhoA activity is generally a negative regulator of dendritic arborization, spine density, and spine growth.

### Rac1

While RhoA serves as a negative regulator, Rac1 works as a positive regulator in dendritic arborization, spine formation, and spine morphology. CA Rac1 increases dendritic complexity in *Xenopus* retinal ganglion cells and rat primary cortical neurons, while DN Rac1 results in a significant reduction in dendritic length and branching in *Xenopus* retinal ganglion cells, rat primary cortical neurons, *Drosophila* mushroom body neurons, and mouse cortical pyramidal neurons (Threadgill et al., 1997; Ruchhoeft et al., 1999; Hayashi et al., 2002; Ng et al., 2002). Rac1 activity also increases growth cone development and neurite growth (Stankiewicz and Linseman, 2014). However, these findings are likely dependent on the specific neuron type, as studies in some systems have shown that CA and DN Rac1 have little to no effect on dendrite length and branching (Luo et al., 1994, 1996; Nakayama et al., 2000). These results may also highlight that Rac1 must cycle between its active and inactive forms in order to function properly. Furthermore, CA Rac1 decreases spine size and increases spine density in mice Purkinje cells, mouse cortical and hippocampal neurons, and rat hippocampal neurons (Luo et al., 1996; Nakayama et al., 2000; Tashiro et al., 2000; Pilpel and Segal, 2004). Interestingly, these same experiments have shown that the presence of CA Rac1 causes a distinct spine morphology—these spines form multiple smaller protrusions that look like miniature spines, which often form double synapses (Govek et al., 2005). Conversely, DN Rac1 decreases spine density, increases spine length, increases spine head width, but impairs spine stability and head motility (Nakayama et al., 2000; Tashiro and Yuste, 2004; Hedrick et al., 2016). Taken together, Rac1 is generally a positive regulator in dendritic arborization, spine formation, and spine maintenance.

### Cdc42

Like Rac1, Cdc42 is a positive regulator of dendritic arborization, spine formation, and spine morphology, though Cdc42 generally has less pronounced effects relative to Rac1. CA Cdc42 increases branching in *Xenopus* optic tectal neurons and increases dendritic growth in chick spinal neurons, while DN Cdc42 reduces the number of dendrites and dendritic length in *Xenopus* retinal ganglion cells mouse pyramidal neurons (Ruchhoeft et al., 1999; Kuhn et al., 2000; Li et al., 2000). Cdc42 activity also leads to increased growth cone development and neurite growth (Stankiewicz and Linseman, 2014). While there have been some conflicting studies in which Cdc42 occasionally appears to negatively regulate dendritic arborization, these discrepancies may again be caused by differences in model systems and the inability of Cdc42 to cycle between its active and inactive states. Furthermore, Cdc42 is still thought to be a positive regulator because many of its upstream regulators increase dendritic arborization (Govek et al., 2005). The role of Cdc42 in spine formation and morphology is less studied and less well understood than those of RhoA and Rac1, but some findings suggest Cdc42 may act as a positive regulator in this case as well. Decreased Cdc42 activity causes a reduction in pyramidal neuron spine density in mice and *Drosophila* (Cheng et al., 2003; Scott et al., 2003). Furthermore, Cdc42 inhibition was found to impair spine maintenance (Murakoshi et al., 2011). However, in other cases, neither CA nor DN Cdc42 affects spine density or morphology (Tashiro et al., 2000). Taken together, these studies suggest that Cdc42 is generally a positive regulator of dendritic arborization, spine formation, and spine maintenance.

While this review focuses on the cytoskeletal functions of small Rho GTPases in neuron development with relevance to delta-catenin as a modulator, more comprehensive reviews describe detailed mechanisms of these GTPases in many aspects of neuron development (Govek et al., 2005; Stankiewicz and Linseman, 2014).

## Delta-Catenin Modulates Rho GTPase Activity

Delta-catenin has been shown to modulate small Rho GTPase activity—specifically the Rho GTPases RhoA, Rac1, and Cdc42 (Braga, 2000; Abu-Elneel et al., 2008; Ghose et al., 2015). Several studies have indicated that delta-catenin expression correlates with Rho inhibition, Rac1 activation, and Cdc42 activation (Martinez et al., 2003; Abu-Elneel et al., 2008; Kim et al., 2008; Gu et al., 2009; Ghose et al., 2015).

### Delta-Catenin in the Cytoplasm

**FIGURE 2:**
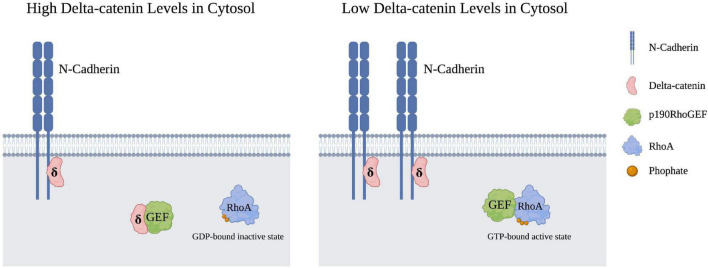
Cytosolic RhoA is inhibited upon the sequestration of p190RhoGEF by cytosolic delta-catenin. An increase in membrane-bound N-cadheren levels in turn lowers delta-catenin presence in the cytosol, such that p190RhoGEF can now transition RhoA from its GDP-bound inactive state to its GTP-bound active state. Created with https://BioRender.com.

While delta-catenin has been shown to modulate the activity of all three aforementioned Rho GTPases, the mechanism underlying delta-catenin’s interaction with RhoA is the most well-characterized. Delta-catenin overexpression has been shown in several instances to inhibit RhoA, which is associated with increased dendritic branching (Martinez et al., 2003; Abu-Elneel et al., 2008; Kim et al., 2008; Gu et al., 2009). More specifically, higher cytosolic levels of delta-catenin inhibit RhoA activity (Kim et al., 2008). Increased cytosolic levels of delta-catenin have been shown to sequester p190RhoGEF, which in turn prevents RhoA from cycling to its GTP-bound active state, as represented in Figure 2 (Kim et al., 2008). Furthermore, delta-catenin requires phosphorylation at T454 by Akt in order to sequester p190RhoGEF (Kim et al., 2008). Because Akt phosphorylation of T454 does not alter Rac1 or Cdc42 activity, delta-catenin’s interactions with RhoA likely occur through a different mechanism than Rac1 or Cdc42 (Kim et al., 2008). Furthermore, p190RhoGEF sequestration and RhoA inhibition by delta-catenin may be modulated by manipulating N-cadherin levels. When N-cadherin levels are elevated, more delta-catenin becomes associated with the N-cadherin-catenin complex which decreases the cytosolic levels of delta-catenin, and in turn, decreases delta-catenin’s cytosolic ability to sequester p190RhoGEF (Gilbert and Man, 2016). Thus, one model is that elevated N-cadherin levels, through increased membrane association of delta-catenin, result in raised RhoA activity and therefore decreased dendritic arborization.

It should be noted that while delta-catenin’s interaction with p190RhoGEF to inhibit RhoA activity is currently the major model that points to a mechanism, delta-catenin may regulate RhoA and other small Rho GTPases through unstudied mechanisms. This is supported by the fact that p120-catenin is known to modulate small Rho GTPases through various mechanisms in which it can associate with GEFs, GAPS, or act as a GDI by binding directly to RhoA (Niessen and Yap, 2006; Anastasiadis, 2007). As such, further studies are needed to elucidate delta-catenin’s potential mechanisms of small Rho GTPase modulation.

RhoA inhibition by delta-catenin may be cell-type and cell-compartment specific. For example, the overall RhoA activity levels in neurons were not altered in homozygous and heterozygous delta-catenin “N-term” mice, which are mice where the generated delta-catenin polypeptide lacks the central-Armadillo and C-terminal tail domains required for its localization and much of its function (Yuan and Arikkath, 2017). On the other hand, in expression studies, the phosphorylation state of delta-catenin’s C-terminal PDZ-binding motif has a significant impact on the activity of RhoA situated in dendrites, if not RhoA in the soma (Baumert et al., 2020). Thus, the response of RhoA to delta-catenin presence may depend on the cellular compartment in question, as well as phospho-states of delta-catenin that influence its protein associations.

While less well-understood, delta-catenin has been indicated to alter Rac1 and Cdc42 activity as well. Delta-catenin increased Rac1 and Cdc42 activity in lymphatic endothelial cells and Chinese hamster ovary cells (Abu-Elneel et al., 2008; Ghose et al., 2015). Furthermore, a dominant negative Rac1 construct was able to inhibit the increased spine density caused by delta-catenin (Abu-Elneel et al., 2008). While neurons from delta-catenin N-term mice did not exhibit lower levels of Rac1 than neurons from wild-type mice (Arikkath et al., 2008), delta-catenin’s ability to alter Rac1 activity levels may be cell-type and cell-compartment dependent. Measuring global levels of small Rho GTPase activity may not detect key changes in the activity of subpopulations of GTPases, such as distinctions in the activity of Rho GTPase in neurites vs. the cell body. Biosensors, that permit visualization of subcellular small Rho GTPase activity, may be useful in future studies to address this issue (Hodgson et al., 2010; Kim et al., 2019).

### Delta-Catenin at Adherens Junctions

At adherens junctions, delta-catenin binds to the cytoplasmic juxta-membrane region of transmembrane classical cadherins, which engage in cell-cell adhesion and signaling (Lu et al., 1999). In mammalian neurons, N-cadherin is the most widely distributed of the classical cadherins (Yagi and Takeichi, 2000). Delta-catenin is thought to stabilize the cadherin-catenin complex. In delta-catenin N-term mice, there is a significant reduction in N-cadherin, beta-catenin, and alpha-catenin protein levels (Fannon and Colman, 1996; Benson and Tanaka, 1998; Israely et al., 2004), which given their linkage to the actin cytoskeleton, participate in conferring adhesive and motility related properties to adherens junctions. This dependence indicates that delta-catenin’s presence is crucial for catenin-cadherin complex stability. While there is currently no evidence that delta-catenin directly interacts with Rho GTPases while bound in the cadherin-catenin complex, N-cadherin-dependent adhesion has been shown to stimulate RhoA activity and decrease Rac1 and Cdc42 activity in C2C12 myoblasts (Charrasse et al., 2002). Delta-catenin’s direct or indirect modulation of small Rho GTPases when bound to cadherins might be suggested by analogy to p120-catenin, which has been more intensively studied in non-neuronal systems (Niessen and Yap, 2006; Anastasiadis, 2007). For example, when associated with N-cadherin, it is conceivable that delta-catenin dissociates briefly to interact with effector molecules of small Rho GTPases before rebinding to N-cadherin. Thus, in addition to cytoplasmic roles, delta-catenin’s modulation of Rho GTPase activity may include doing so through relationships with the N-cadherin-catenin complex at adherens junctions.

### Delta-Catenin’s C-Terminal Binding Partners

**FIGURE 3:**
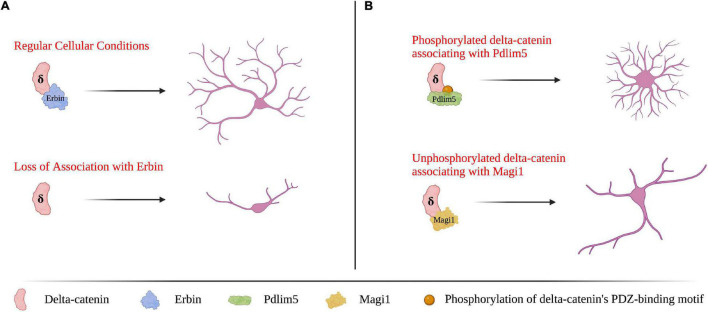
Delta-catenin’s PDZ-binding motif at its C-terminus is essential for shaping dendrite morphology *via* binding with PDZ-domain proteins. **(A)** Delta-catenin requires Erbin to bind its PDZ-binding motif for delta-catenin to promote dendritic arborization. In the absence of delta-catenin association with Erbin (e.g., Erbin depletion), delta-catenin cannot localize properly which results in decreased dendrite length and branching. Based on findings from Arikkath et al. (2008). **(B)** When delta-catenin’s PDZ-binding motif is phosphorylated, delta-catenin favors binding Pdlim5, which promotes dendritic branching. Conversely, when delta-catenin’s PDZ-binding motif is unphosphorylated, delta-catenin has the propensity to bind Magi1, which promotes dendritic lengthening. Based on findings from Baumert et al. (2020). Neuron morphology is not intended to be compared between panels **(A,B)**, as they are based on findings from separate studies. Created with https://BioRender.com.

Delta-catenin’s morphological influence in neurons is due to interactions that occur with effectors at its various domains. One such domain is the PDZ-binding motif, which is present at delta-catenin’s C-terminus and enables it to bind to select protein partners that contain PDZ domains. Although the interactions involving delta-catenin’s binding partners are still being studied, delta-catenin’s PDZ domain interactions have been found necessary for dendritic spine morphology in developing neurons (Yuan et al., 2015). While expression of a full-length delta-catenin construct can rescue spine density and architecture following shRNA-mediated knockdown in developing neurons, a delta-catenin construct that is missing its small C-terminal PDZ motif is unable to do so (Yuan et al., 2015). A notable interaction at delta-catenin’s PDZ motif is with Erbin (Laura et al., 2002). Erbin regulates the localization of delta-catenin, which influences delta-catenin’s effects on dendrite morphology (Arikkath et al., 2008). Furthermore, phosphorylation of delta-catenin’s PDZ-binding motif influences its binding partners and thus functions. In primary rat hippocampal neurons, the phosphorylation state of S1242 and S1245 (corresponding to the –3 and –6 residues counting back from delta-catenin’s C-terminal valine), determines whether delta-catenin binds to Magi1 or Pdlim5 (Baumert et al., 2020). When these serine residues are unphosphorylated, delta-catenin binds with Magi1, which promotes dendritic elongation; conversely, when the same serine residues are phosphorylated, delta-catenin binds Pdlim5, which promotes dendritic branching (Baumert et al., 2020). Selected functional outcomes of delta-catenin’s PDZ domain-containing binding partners are shown in Figure 3. Delta-catenin also has associations within its C-terminal domain that are upstream of its above-noted PDZ binding motif, and that likewise influence dendrite morphogenesis (Lee et al., 2015). Some notable interactions are with cortactin and thereby with actin (Kim et al., 2002; Martinez et al., 2003). When the tyrosine that resides ninety-nine residues upstream from delta-catenin’s C-terminus (Y1126) is unphosphorylated, delta-catenin associates with cortactin, which promotes neurite elongation but not branching in PC12 cells and hippocampal neurons (Martinez et al., 2003). While delta-catenin’s PDZ-binding motif has not been shown to directly influence Rho GTPase activity, several PDZ-domain proteins are known to both function and physically interact with Rho GTPases, including members of the PDZ/LIM (also known as Pdlim or Enigma protein) family, the Magi family, Ptpl family, and the protein PIST (Saras et al., 1997; Neudauer et al., 2001; Sakurai et al., 2006; te Velthuis and Bagowski, 2007; Zhang et al., 2007; Kozasa et al., 2011; Liu et al., 2015). Thus, delta-catenin’s PDZ-binding motif may serve as another method to indirectly influence Rho GTPase activity.

## Functional Roles of Delta-Catenin

### Dendrites, Spines, and Synapses

Delta-catenin has several known functional roles in neurons, including regulating dendrite arborization and spine morphology (Abu-Elneel et al., 2008; Yuan et al., 2015). Given the roles of small Rho GTPases in cytoskeletal reorganization, these functional roles are likely due to delta-catenin’s ability to modulate RhoA, Rac1, and Cdc42. Upon overexpression, delta-catenin increases dendritic protrusion density in primary hippocampal neurons (Abu-Elneel et al., 2008; Kim et al., 2008; Brigidi et al., 2014; Baumert et al., 2020). Conversely, delta-catenin loss *via* shRNA-mediated knockdown reduces the dendritic arbor in primary hippocampal neurons (Arikkath et al., 2008; Baumert et al., 2020). Reduced dendritic length and arborization upon delta-catenin knockdown have been confirmed *in vivo via* N-term mice (Israely et al., 2004; Arikkath et al., 2008). Based upon two-photon imaging of brain tissue from these N-term mice, delta-catenin knockdown does not affect the dendritic arbor before 5 weeks. However, the amount of distal dendritic branches in neurons decreased in mice after that time (Matter et al., 2009). Based on these morphological effects observed following delta-catenin overexpression and knockdown, delta-catenin may possess distinct roles during different stages of development, for example, promoting dendrite arborization during development and stabilization in adulthood (Yuan and Arikkath, 2017).

While delta-catenin is known to influence dendritic spine morphology, studies have shown somewhat contradicting results. In primary hippocampal neurons, the overexpression of delta-catenin increases spine and synapse density (Abu-Elneel et al., 2008; Turner et al., 2015). Furthermore, siRNA-mediated knockdown of delta-catenin in primary hippocampal neurons decreases spine and synapse density (Abu-Elneel et al., 2008). However, similar studies in neurons from N-term mice have shown differing results. In these studies, the endogenous expression of N-term delta-catenin leads to an increase in synapse and spine density (Arikkath et al., 2009; Brigidi et al., 2014). There are several possible explanations for the seeming inconsistencies among studies of delta-catenin’s effects on spine morphology. For example, a genuine knock-out of delta-catenin still needs to be generated and assessed, since delta-catenin N-term, even while lacking delta-catenin’s central-Armadillo and C-terminal-tail domains, may have unknown (e.g., dominant-active or –negative) properties. Further, differences in neuron or glial cell density in culture, or in which splice variant of delta-catenin is expressed, may influence how delta-catenin interacts with its binding partners to influence spine density (Yuan and Arikkath, 2017). Subsequent work will be needed to resolve these puzzles. In all cases, given delta-catenin’s interactions with Rho GTPases and the impact that Rho GTPases have on dendrite and spine morphology, delta-catenin’s morphological effects are likely to result in significant part from functional interactions with small Rho GTPases.

## Pathological Associations of Delta-Catenin

### Neurological Pathologies

Consistent with delta-catenin’s influence on dendrite and spine morphogenesis and maintenance in neurons through interactions with small Rho GTPases, abnormalities in delta-catenin have been implicated in several neurological pathologies. A summary of delta-catenin’s associated pathologies is shown in Table 1. In fact, delta-catenin was first identified in a screen to identify binding partners with presenilin 1 (PS1), which is the most commonly mutated gene in AD (Zhou et al., 1997). Further investigation showed that delta-catenin recruits PS1 to cadherins, which inhibits amyloid-beta production (Zhou et al., 1997; Kouchi et al., 2009). A genome-wide association study (GWAS) found multiple delta-catenin mutations associated with AD, including SNP (single nucleotide polymorphism) rs17183619 and a rare missense mutation (G810R), both of which significantly increase amyloid-beta in neuronal cell culture (Jun et al., 2012). This same study also found that cortical cataracts may predict future AD and that both are associated with the same delta-catenin mutations (Jun et al., 2012). Small Rho GTPases have also been extensively implicated in AD, which has been reviewed elsewhere (Bolognin et al., 2014). In AD, synaptic degeneration is a significant cause of cognitive impairment (Arendt, 2009). As small Rho GTPases modulate dendritic spine density as described above, they have well-established involvement in synaptic degeneration associated with AD. While interactions between delta-catenin and small Rho GTPases have not been studied in the context of AD, it is conceivable that such interactions may play a role in AD progression. As mentioned above, delta-catenin expression inhibits RhoA and activates Rac1 and Cdc42, all of which generally increase synaptic density. Thus, loss of function mutations in delta-catenin would increase RhoA activity while decreasing that of Rac1 and Cdc42. This would theoretically decrease spine density, which could contribute to the synaptic degeneration associated with AD. However, as this proposed mechanism is purely speculative, further studies are required to determine if the delta-catenin mutations associated with AD alter small Rho GTPase activity and spine morphology as predicted.

**TABLE 1:** Summary of delta-catenin’s associated pathologies.

Pathology	Delta-catenin modifications	References
**Neurological pathologies**		
Alzheimer’s Disease	SNP rs17183619 found in GWAS. Rare missense mutation G810R found in GWAS.	Jun et al., 2012
Intellectual Disability	CNVs found in a case study (partial deletions). Translocation breakpoint found in a case study. Deletion of exons 12–18 found in a case study.	Hofmeister et al., 2015; Belcaro et al., 2015
Cri-du-chat Syndrome	CNV found in a case study (partial duplication + partial deletion). Functional deletion found in delta-catenin N-term mice. Hemizygous deletion of 5p15.2 found in case studies.	Israely et al., 2004; Sardina et al., 2014
Autism Spectrum Disorder	CNV found in case studies (partial duplication + partial deletion). Mutations G274C, G34S, Q507P, R713C, T862M found in a case study.	Turner et al., 2015
Schizophrenia	CNV found in a case study (duplication). Several CNVs found in GWAS. Several SNPs found in GWAS (strongest association rs4524507).	Stefansson et al., 2008; Vrijenhoek et al., 2008; Nivard et al., 2014
**Non-neurological pathologies**		
Cancer	Multiple mutations found in GWAS, samples, and case studies.	Lu et al., 2016
Myopia	Multiple SNPs found in case studies and meta-analysis.	Lu et al., 2011; Yu et al., 2012; Liu and Zhang, 2014
Malaria Resistance	An environmental correlation analysis showed that delta-catenin contributes to malaria resistance.	Mackinnon et al., 2016

Alterations of delta-catenin are also known to cause intellectual and cognitive dysfunction. N-term mice demonstrated severe spatial learning deficiency compared to normal mice (Israely et al., 2004). These same mice also had synaptic plasticity and composition deficits and accordingly displayed reduced N-cadherin and PSD95 levels (Israely et al., 2004). Studies in humans have confirmed delta-catenin’s link to intellectual disability by investigating mutations in patients with said disabilities. Delta-catenin’s lowered or aberrant expression resulting from copy number variations (CNVs), delta-catenin microdeletions, and a breakpoint of intron 9 on chromosome 5 of delta-catenin have all been seen in patients with intellectual disability and dyslexia (Belcaro et al., 2015; Hofmeister et al., 2015). Similar to the possible mechanism described in the context of AD, the synaptic plasticity deficits associated with lowered delta-catenin expression may be due to the corresponding alterations of small Rho GTPase activity.

Abnormal delta-catenin expression has also been correlated with Cri-du-chat syndrome. Hemizygous delta-catenin loss has been found in children with Cri-du-chat syndrome that are characterized by severe intellectual disability, although deletions of the entire 5p15.2 region of the chromosome encompass genes other than delta-catenin as well. However, the partial deletion of delta-catenin has also been associated with mild cases of Cri-du-chat syndrome (Sardina et al., 2014), which helps confirm its association with the disease in general. While small Rho GTPases have not been extensively studied in the context of Cri-du-chat syndrome, it is thought that the cognitive effects of Cri-du-chat may be in part due to alterations of small Rho GTPase activity caused by delta-catenin loss of function (Correa et al., 2019).

Delta-catenin haploinsufficiency has also been linked to autism spectrum disorder (ASD), which is a neurodevelopmental disorder that affects behavioral and social attributes (Schieve et al., 2012). Multiple mutations in delta-catenin (G274C, G34S, Q507P, R713C, T862M) were found to be associated with ASD, which were then functionally confirmed in ASD patients and zebrafish (Turner et al., 2015). This study further found that these mutations are loss-of-function mutations that affect Wnt signaling (Turner et al., 2015). Though studies on the roles of small Rho GTPases in ASD are limited, mutations in several genes that affect small Rho GTPase activity have been implicated (Lin et al., 2016; Tian et al., 2018; Guo et al., 2020).

There have additionally been several mutations identified that appear relevant to delta-catenin contributions in schizophrenia. GWAS studies have shown that both CNVs and SNPs of delta-catenin are associated with schizophrenia (Stefansson et al., 2008; The International Schizophrenia Consortium, 2008; Nivard et al., 2014). Furthermore, a patient with schizophrenia had a CNV that caused a breakpoint in delta-catenin (Vrijenhoek et al., 2008). Interestingly, GWAS studies have also shown SNPs on delta-catenin that are associated with anxiety and depression (Nivard et al., 2014). Recent studies in delta-catenin transgenic mice have confirmed that higher levels of delta-catenin are correlated with reduced anxiety and improved memory (Ryu et al., 2019).

### Non-neurological Pathologies

Despite a relatively diminished presence in non-neuronal tissue, delta-catenin has been implicated in several non-neurological diseases. Both delta-catenin and small Rho GTPases have been well implicated in multiple cancer types. Given that the roles of delta-catenin and small Rho GTPases in cancer have been reviewed in detail previously (Lu, 2010; Haga and Ridley, 2016; Lu et al., 2016; Svensmark and Brakebusch, 2019), and that the focus of this review is upon neurons, only a brief overview is presented here. To date, according to the Sanger Catalog of Somatic Mutations in Cancer, there have been 4,161 unique samples with delta-catenin mutations from 41,032 total samples. Most cancers exhibit increased expression of delta-catenin, except for central nervous system cancers, which exhibit reduced delta-catenin expression (Lu et al., 2016). Point mutations and CNVs on delta-catenin have been reported in the skin, large intestine, stomach, lung, cervical, bladder, esophagus, urinary tract, soft tissue, prostate, liver, breast, ovarian, and endometrial cancers (Burger et al., 2002; Zheng et al., 2004; Lu et al., 2005, 2009; Huang et al., 2006). In recent years, there has been an increase in studies regarding delta-catenin’s roles in prostate cancer, with the proposal that delta-catenin may prove to be a therapeutic target (Shrestha et al., 2018; Zhang et al., 2018; Zhou et al., 2019; Li et al., 2020; Shen et al., 2021; Chen et al., 2022). While both delta-catenin and small Rho GTPases have been implicated in cancer, studies linking the two are limited. That being said, delta-catenin has been shown to modulate lymphangiogenesis, proposed to be *via* consequent effects upon small Rho GTPases (Ghose et al., 2015). Aside from its roles in cancer, delta-catenin has been implicated in several genomic studies with potential roles in myopia and malaria resistance, each being diseases where the altered activity of small Rho GTPases has been implicated (Lam et al., 2008; Lu et al., 2011; Atkinson et al., 2012; Yu et al., 2012; Band et al., 2013; Liu and Zhang, 2014; Wang et al., 2014; Mackinnon et al., 2016; Yuan et al., 2018; Paone et al., 2020). However, delta-catenin’s specific roles in these diseases have yet to be thoroughly grounded. That is, future studies may consider the interplay between delta-catenin and small Rho GTPases in the context of these diseases.

## Conclusion

Although the field has advanced delta-catenin as an established modulator of small Rho GTPases in neuronal morphology and function, understanding delta-catenin’s more defined mechanisms of action will in many cases require further work. In general, delta-catenin expression inhibits RhoA and activates Rac1 and Cdc42 (Martinez et al., 2003; Abu-Elneel et al., 2008; Kim et al., 2008; Gu et al., 2009; Ghose et al., 2015). However, evidence indicates that these interactions may depend on a variety of other factors, including the subcellular compartment under consideration (e.g., neurite vs. soma), type of cell, tissue, species, or the phosphorylation or splice-isoform status of delta-catenin’s various domains (i.e., binding partners). Of delta-catenin’s known effects on small Rho GTPase activity, only its inhibition of RhoA has been studied to the point of a proposed mechanism. That mechanism is likely to represent but one of many distinct functional interactions of delta-catenin with RhoA, analogous to p120-catenin having more than one mechanism supporting its functional relationships with RhoA (Niessen and Yap, 2006; Anastasiadis, 2007). Further studies are needed to elucidate a mechanism for how delta-catenin activates Rac1 and Cdc42, with possibilities that may again be suggested from the studies of other structurally related catenins. Among variables to consider in such an undertaking, we will require a better understanding of upstream factors that instruct outcomes including delta-catenin’s phosphorylation status, isoform usage, and sub-cellular localization. Various techniques are available to do this, such as protein modifications to mimic phosphorylation or dephosphorylation, as well as existing biosensors to examine small Rho GTPase activity at the subcellular level. As future studies in the field elucidate mechanisms acting upon delta-catenin that in turn modulate small Rho GTPases, our understanding of synaptic formation, learning, and memory will progress.

While both delta-catenin and small Rho GTPases have been implicated in many of the same pathologies, links between the two in the context of disease have rarely been studied, particularly in neurological pathologies (Govek et al., 2005; Lu et al., 2016). As dendritic arbor and spine abnormalities are seen in many of these cases, there are likely contributing interactions between delta-catenin and small Rho GTPases. However, further studies are required to determine if abnormal delta-catenin expression affects small Rho GTPase activity accordingly in disease models. This could be done by investigating how small Rho GTPase activity changes in diseases with mutant delta-catenin (i.e., specific delta-catenin mutations that have been correlated with disease) compared to wild-type delta-catenin. Connecting delta-catenin to small Rho GTPase activity in disease models could help further our mechanistic understanding of several debilitating diseases, such as cancer, AD, ASD, and Cri-du-chat syndrome.

## Author Contributions

MD and PM wrote and finalized the manuscript. MD and YS drafted and revised the figures. PM guided the writing process. All authors contributed to the article and approved the submitted version.

## Conflict of Interest

The authors declare that the research was conducted in the absence of any commercial or financial relationships that could be construed as a potential conflict of interest.

## Publisher’s Note

All claims expressed in this article are solely those of the authors and do not necessarily represent those of their affiliated organizations, or those of the publisher, the editors and the reviewers. Any product that may be evaluated in this article, or claim that may be made by its manufacturer, is not guaranteed or endorsed by the publisher.
